# Cross-talk between NLRP3 and AIM2 inflammasomes in macrophage activation by LPS and titanium ions

**DOI:** 10.1186/s10020-025-01290-7

**Published:** 2025-06-09

**Authors:** Ana Belén Carrillo-Gálvez, José Antonio Guerra-Valverde, Miguel Padial-Molina, Andrea Martínez-Cuevas, Darío Abril-García, Allinson Olaechea, Natividad Martín-Morales, Francisco O’Valle, Pablo Galindo-Moreno, Federico Zurita

**Affiliations:** 1 Department of Oral Surgery and Implant Dentistry, School of Dentistry, University of Granada, Granada, Spain; 2 Instituto de Investigación Biosanitaria (ibs) de Granada, Granada, Spain; 3 Clinical Medicine and Public Health, University of Granada, Granada, Spain; 4 Department of Genetics, University of Granada, Granada, Spain; 5 GENYO. Centre for Genomics and Oncological Research: Pfizer, University of Granada/Andalusian Regional Government PTS Granada - Avenida de la Ilustración, Granada, 114 - 18016 Spain; 6 Department of Pathology, School of Medicine, University of Granada, Granada, Spain; 7 Institute of Biopathology and Regenerative Medicine (IBIMER, CIBM), University of Granada, Granada, Spain; 8 Institute of Biotechnology, University of Granada, Granada, Spain

**Keywords:** Inflammation, Peri-implant disease, Titanium, NLRP3, AIM2, IL-1β, Cell signaling

## Abstract

**Background:**

Periodontitis and peri-implantitis are chronic inflammatory diseases that contribute to tissue destruction and bone loss. Periodontitis is triggered by pathogenic bacteria, while peri-implantitis also involves metallic particles, which increase the inflammatory response. Both conditions are linked to the activation of inflammasomes, such as NLRP3 and AIM2, which facilitate the release of pro-inflammatory cytokines like IL-1β and IL-18 and induce pyroptosis. This study aims to investigate the activation of NLRP3 and AIM2 inflammasomes in macrophages exposed to bacterial and metallic components, as well as to explore the potential interplay between these two signaling pathways.

**Methods:**

Human THP-1-derived macrophages were treated with bacterial lipopolysaccharide (LPS) and titanium ions to evaluate inflammasome activation. IL-1β secretion, ROS production, mitochondrial DNA release and pyroptosis were assessed. Additionally, macrophages deficient in NLRP3 and AIM2 were used to examine the roles of these inflammasomes in inflammatory responses.

**Results:**

LPS and titanium ions synergistically activated NLRP3, resulting in increased IL-1β secretion, ROS production, and pyroptosis. Under these conditions, AIM2 was indirectly activated, as indicated by elevated mitochondrial DNA release. Notably, AIM2 expression was reduced in wild-type macrophages treated with LPS and titanium ions compared to LPS alone, however, in NLRP3-deficient cells, AIM2 expression was increased following LPS and titanium ions treatment. This upregulation of AIM2 in NLRP3-deficient cells was further reduced by ROS inhibition, which decreased mitochondrial DNA release. Additionally, NLRP3 knockout had a more pronounced effect on reducing IL-1β secretion and pyroptosis compared to AIM2 knockout, indicating a greater role of NLRP3 in these inflammatory responses.

**Conclusions:**

This study demonstrates that bacterial and metallic components drive the activation of both NLRP3 and AIM2 inflammasomes in macrophages, highlighting their roles in the inflammatory responses associated with periodontitis and peri-implantitis. The findings reveal a regulatory relationship between NLRP3 and AIM2, where the absence of one inflammasome can enhance the activity of the other. These results provide new insights into the mechanisms underlying inflammasome-mediated inflammation and suggest potential therapeutic targets for managing inflammatory diseases.

## Background

Chronic inflammation is a pathological process underlying numerous diseases and disorders, including periodontal and peri-implant diseases. Periodontitis is a multifactorial disease primarily induced by pathogenic bacteria that disrupt the balance of the oral microbiome, triggering exacerbated immune responses that lead to the destruction of periodontal tissues and bone loss (Papapanou et al. 2018). It is a highly prevalent disease that affects around 60% of the total adult population (Paul et al. 2021). On the other hand, peri-implantitis, a common complication of dental implants, is characterized by chronic inflammation of the peri-implant tissue accompanied by progressive bone resorption. This condition is not only associated with the accumulation of bacterial biofilms but also with the release of metal particles from the implant surface (Derks et al. 2016). Titanium, widely used in dentistry for its biocompatibility, can exert pro-inflammatory effects under certain conditions. Released titanium particles act as danger-associated molecular patterns (DAMPs), triggering inflammatory responses by interacting with immune cells (Bressan et al 2019; Suárez-López del Amo et al. 2017).

The inflammatory process can be triggered through the activation of the inflammasome pathway. Inflammasomes are multiprotein complexes that facilitate the proteolytic cleavage, maturation, and release of pro-inflammatory cytokines, such as interleukin 1β (IL-1β) and 18 (IL-18). Additionally, inflammasomes also promote an inflammatory form of cell death known as pyroptosis through the activation of Gasdermin-D (GSDMD) (Marchesan et al. 2019). One of the most studied inflammasomes is NLRP3 (NLR family pyrin domain containing 3) which recognizes and is activated by DAMPs and PAMPs (pathogen-associated molecular patterns) (Swanson et al. 2019). AIM2 (absent in melanoma 2) inflammasome, however, is only able to recognize and be activated by double-stranded DNA (dsDNA) (Fernandes-Alnemri et al. 2009).

Inflammasomes are primarily expressed in cells of the innate immune system, mainly macrophages, neutrophils and dendritic cells (Honda et al. 2023; Kumari et al. 2020). Macrophages play a crucial role in periodontitis and peri-implantitis as they act as mediators of inflammation and tissue destruction and repair due to their ability to adopt a pro-inflammatory (M1) or anti-inflammatory (M2) phenotype depending on the signals received (Mo et al. 2024; Li et al. 2024). There are few studies that have analyzed *in vitro* the activation of NLRP3 in macrophages in response to triggers of peri-implantitis, such as metal ions or particles. These studies suggest that the combination of bacterial and metallic components induces an enhanced inflammatory response in macrophages (Pettersson et al. 2017; Pettersson et al. 2022). In the case of AIM2, there are no studies that have examined *in vitro* its activation in macrophages within the context of periodontitis or peri-implantitis.

This report emphasizes how bacterial components (LPS) and metallic elements (titanium ions) drive the activation of NLRP3 inflammasome and indirectly stimulate AIM2 activation. We further examine the effects of NLRP3 and AIM2 deficiency on the inflammatory response, revealing that the absence of NLRP3 alters AIM2 activation, potentially indicating a compensatory mechanism employed by the cell to compensate the loss of NLRP3. In summary, this study highlights the critical role of both inflammasomes in periodontitis and peri-implantitis associated inflammation and reveals a mutual regulatory relationship between these signaling pathways.

## Materials and methods

### Cell culture

The human monocytic cell line THP-1 was obtained from the “Centro de Instrumentación Científica (CIC)” (University of Granada) and cultured in RPMI 1640 (Biowest) supplemented with 10% heat-inactivated fetal bovine serum (FBS) (Sigma-Aldrich), 50 µM of 2-Mercaptoethanol and 100 U/mL of penicillin/streptomycin (Gibco). THP-1 cells were incubated and maintained under 21% O_2_/5% CO_2_ at 37°C and were routinely tested for mycoplasma contamination.

### Genome editing of THP-1

Generation of NLRP3 or AIM2-knockout (KO) THP-1 cells was performed using lentiviral vectors encoding a guide RNA (gRNA) specific for each gene, as well as the Cas9 (Crispr associated protein 9) protein. A nonspecific gRNA was used as a control. The efficiency of each gRNA was previously validated by our research group (Carrillo-Gálvez et al. 2024). Lentiviral particles were provided by Vector Builder Company (VectorBuilder Inc.) with a viral titer > 10^9^ infectious particles/mL. The sequence of the different gRNAs is detailed below:

- NLRP3 gRNA sequence: CGGTCCTATGTGCTCGTCAA.
- AIM2 gRNA sequence: TCTTGGGTCTCAAACGTGAA.
- CTRL gRNA sequence: GTGTAGTTCGACCATTCGTG.

### Transduction of THP-1

For THP-1 transduction, 0.2 × 10⁶ cells were incubated with lentiviral particles (MOI = 35) in the presence of 4 µg/mL Polybrene to enhance transduction efficiency. After 5 h of incubation at 37°C, cells were centrifuged, cultured in fresh medium and maintained in culture for 4–6 days to allow the expression of the transduced constructs. From each edited bulk population (NLRP3 and AIM2), individual clones were subsequently isolated by limiting dilution in 96-well plates, aiming for one cell per well. Wells containing a single visible cell were identified by microscopy, and the selected clones were expanded for approximately two weeks. Cell pellets were then collected and frozen for subsequent validation of gene editing efficiency.

### Verification of CRISPR gene editing efficiency

To check the efficiency of gene editing, genomic DNA was isolated from bulk-edited THP-1 cells and from the different isolated clones using a Quick-DNA Miniprep Kit (Zymo Research). Genomic regions surrounding the CRISPR/Cas9 target site for each gRNA were amplified via PCR using the MyTaq Red Mix, 2X Kit (Bioline). The PCR products were then purified with the DNA Clean & Concentrator-5 Kit (Zymo Research) and subjected to Sanger sequencing using the same primers employed in the PCR. Sequencing data were analyzed using the ICE Software from Synthego (https://ice.synthego.com/#/) comparing each sequence with a control sequence from non-transduced cells. The ICE score indicated editing via non-homologous end joining (NHEJ). The primers used are shown in Table 1.

**Table 1:** Sequence of primers used for PCR and RT-qPCR analyzes

**Primers**		
Gene	Forward sequence	Reverse sequence
NLRP3-KO check	5´-CAGGAAGATGATGTTGGACT-3´	5´-AAGGAAGAAGACGTACACCG-3´
AIM2-KO check	5´-CTTCCCTTGATTCCACCTAT-3´	5´-CTGAGTTTGAAGCGTGTTGA-3´
NLRP3	5´-AGCCCCGTGAGTCCCATTA-3´	5´-ACGCCCAGTCCAACATCATCT-3´
AIM2	5´-ACAGGCCTGGATAACATCACT-3´	5´-ACCGCCCCAGCATTTTGAAT-3´
CASP1	5´-GCCTGTTCCTGTGATGTGGAG-3´	5´-TGCCCACAGACATTCATACAGT-3´
GSDMD	5´-ATGGATGGGCAGATACAGGG-3´	5´-TGCTGCAGGACTTTGTGTTC-3´
GAPDH	5´-AGCTCATTTCCTGGTATGACAAC-3´	5´-TTACTCCTTGGAGGCCATGTG-3´
COX-1	5´-TCTCAGGCTACACCCTAGACCA-3´	5´-ATCGGGGTAGTCCGAGTAACGT-3´
ND-1	5´-CGATTCCGCTACGACCAACT-3´	5´-AGGTTTGAGGGGGAATGCTG-3´
CXCL9	5´-GCTGGTTCTGATTGGAGTGC-3´	5´-GAAGGGCTTGGGGCAAATTG-3´
CXCL10	5´-CGCTGTACCTGCATCAGCAT-3´	5´-CGTGGACAAAATTGGCTTGC-3´
IRF-1	5´-TGACCACAGCAGCTACACAG-3´	5´-CGACTGCTCCAAGAGCTTCA-3´
CCL17	5´-CTTCTCTGCAGCACATCCAC-3´	5´-CAGATGTCTGGTACCACGTC-3´
ALOX15	5´-CAGATGTCCATCACTTGGCAG-3´	5´-CTCCTCCCTGAACTTCTTCAG-3´
MRC1	5´-CGAGGAAGAGGTTCGGTTCACC-3´	5´-GCAATCCCGGTTCTCATGGC-3´

### Immunofluorescence

For immunofluorescence analysis, 50,000 edited or non-edited THP-1 cells were seeded in 24-well plates and treated with 100 ng/mL of phorbol 12-myristate-13-acetate (PMA) (Sigma-Aldrich) for 48 h. Subsequently, cells were fixed with 4% paraformaldehyde (PFA) (Sigma-Aldrich), permeabilized with 0.25% Triton X-100 (Sigma-Aldrich), and blocked with 2% BSA (Bovine Serum Albumin) (Sigma-Aldrich). THP-1 cells were then incubated overnight with primary antibodies against human NLRP3 or AIM2 (Invitrogen and MyBioSource, respectively). The following day, cells were treated with a secondary antibody (Goat anti-Rabbit IgG (H + L) Cross-Adsorbed Secondary Antibody, Alexa Fluor 488, from Invitrogen) for one hour, and Hoechst was used for nuclear counterstaining. Controls were performed using only primary or secondary antibodies. Images were captured using a Nikon Eclipse Ts2 microscope, and fluorescence intensity was quantified using the ImageJ digital image processing software.

### Inflammasome activation in differentiated THP-1 cells

To induce NLRP3 and/or AIM2 expression in THP-1 cells, 0.25 × 10^6^ edited or non-edited THP-1cells were seeded in 24-well plates and treated with 100 ng/mL of PMA to induce an undifferentiated macrophage (M0) phenotype. After 48 h of incubation with PMA, cells were treated with 500 ng/mL of lipopolysaccharide (LPS *E.coli* O111:B4, Sigma-Aldrich) with or without 20 µg/mL of a titanium ion solution (Ti) (Titanium atomic absorption standard solution, Sigma-Aldrich) for 24 h. This Ti concentration falls within the physiological range of patients with peri-implantitis (20–30 µg/mL) (Sharanappa et al. 2024).

### Quantitative PCR (qPCR)

Total RNA was extracted using TRIzol reagent following the manufacturer’s protocol. RNA samples were then reverse transcribed with the PrimeScript^®^ RT Master Mix (Perfect Real Time) (TaKaRa Bio Inc.), and reverse-transcription (RT)-qPCR was conducted using the TB Green Premix Ex Taq (Tli RNase H Plus) (TaKaRa Bio Inc.) on a Real-Time PCR Thermal Cycler qTOWER3 system. The primers employed are listed in Table 1.

### Enzyme-Linked immunosorbent assay (ELISA)

Supernatants from LPS and/or Ti-treated cells were centrifuged at 1,000 x g for 10 min at 4°C. IL-1β protein levels were assessed using the Human IL-1 beta Uncoated ELISA kit (Invitrogen), following the manufacturer’s instructions. Absorbance was measured at 450 nm using an Infinite M200 Pro Microplate Reader, and protein concentration was determined by comparing the values to a standard curve.

### Quantification of lactate dehydrogenase (LDH) release

To analyze lactate dehydrogenase secretion levels, 70,000 edited and unedited THP-1 cells were subjected to the different LPS and Ti treatments explained above. Subsequently, LDH levels were measured using the LDH Cytotoxicity Assay Kit (Assay Genie) according to the manufacturer’s protocol. Absorbance was measured at 450 nm using an Infinite M200 Pro Microplate Reader.

### Intracellular reactive oxygen species (ROS) measurement

Intracellular ROS were measured using the Fluorometric Intracellular ROS Kit (Sigma-Aldrich). For this, edited and non-edited THP-1 cells were treated with 5 mM NAC (*N*-Acetyl-L-cysteine) during two hours and with LPS and/or Ti for six hours. Subsequently, the ROS Detection Reagent was added to the cells, and they were incubated at 37°C for one hour. Fluorescence intensity was then measured at λex = 540 nm/λem = 570 nm using an Infinite M200 Pro Microplate Reader.

### Measurement of cytoplasmic mitochondrial DNA (mtDNA)

For cytoplasmic isolation, 1 × 10^6^ edited and non-edited THP-1 cells were seeded in 6-well plates and subjected to the different treatments explained above for 24 h. Cells were collected, resuspended in 0,5 mL hypotonic buffer (10 mM Tris-HCl pH = 8; 1,5 mM MgCl_2_; 10 mM NaCl and 1 mM DTT) and incubated for 5 min on ice. Then, cells were citoplasmically lysed adding 0,1% NP-40 (IGEPAL, Santa Cruz Biotechnology) and incubated for 20 min on ice. THP-1 cells were centrifuged at 1,000 x g for 5 min, cell supernatants were collected and centrifuged again at 15,000 x g for 15 min. Cell supernatants were stored at −80ºC for subsequent mtDNA purification.

Cytoplasmic mtDNA was purified using a Quick-DNA Microprep Kit (Zymo Research) and qPCR analysis was performed for relative mtDNA quantification. The primers employed are listed in Table 1.

### Ratio M1/M2

Polarization of THP-1 cells toward M1 (pro-inflammatory) or M2 (anti-inflammatory) macrophages was analyzed as follows: THP-1 edited and unedited cells were treated with LPS and/or Ti for 24 h and complementary DNA (cDNA) was obtained as explained previously. Subsequently, qPCR analysis was performed for three M1 macrophage-specific markers (CXCL9, CXCL10, and IRF-1) and for three M2 macrophage-specific markers (CCL17, ALOX15, and MRC1) (Baxter et al. 2020). Finally, the ratio of relative expression levels of M1 genes to M2 genes was calculated. Values > 1 indicate polarization toward M1 macrophages, and values < 1 indicate polarization toward M2 macrophages. The sequence of the primers employed are listed in Table 1.

### Statistical analysis

Statistical analysis was conducted using GraphPad Prism software. Data are presented as the mean ± SD from at least three independent experiments. The normality of the data was assessed using the Shapiro-Wilk test. To compare multiple groups, one-way or two-way analysis of variance (ANOVA) was applied, followed by Tukey’s post-hoc test. A *p*-value ≤ 0.05 was considered statistically significant.

## Results

### Generation of NLRP3 and AIM2 knockout THP-1 cells

**Fig. 1:**
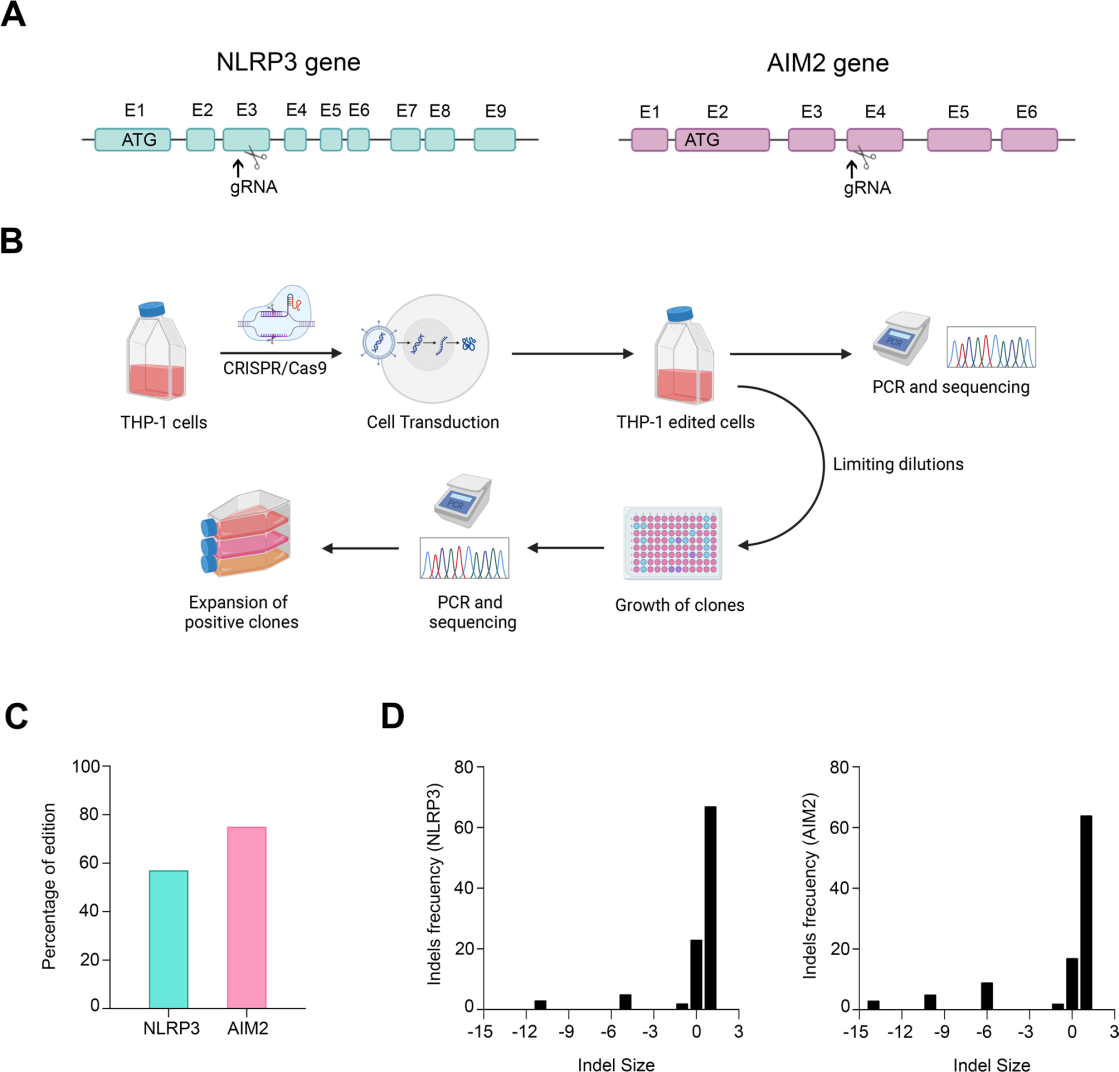
**A** Schematic representation of the NLRP3 (left panel) and AIM2 (right panel) genes. Black arrows indicate the exon where the gRNA is targeted. **B** Representative scheme of the transduction process utilizing lentiviral vectors, accompanied by PCR analysis of genomic DNA to assess gene-editing efficiency. Image generated using BioRender.com. **C** Percentage of edition at the human NLRP3 and AIM2 loci using CRISPR/Cas9, determined through the ICE algorithm. **D** Graphs displaying the indel profiles generated in THP-1 cells using the ICE algorithm. The coordinate zero represents unedited sequences, negative values represent deletions and positive values represent insertions of different length

**Fig. 2:**
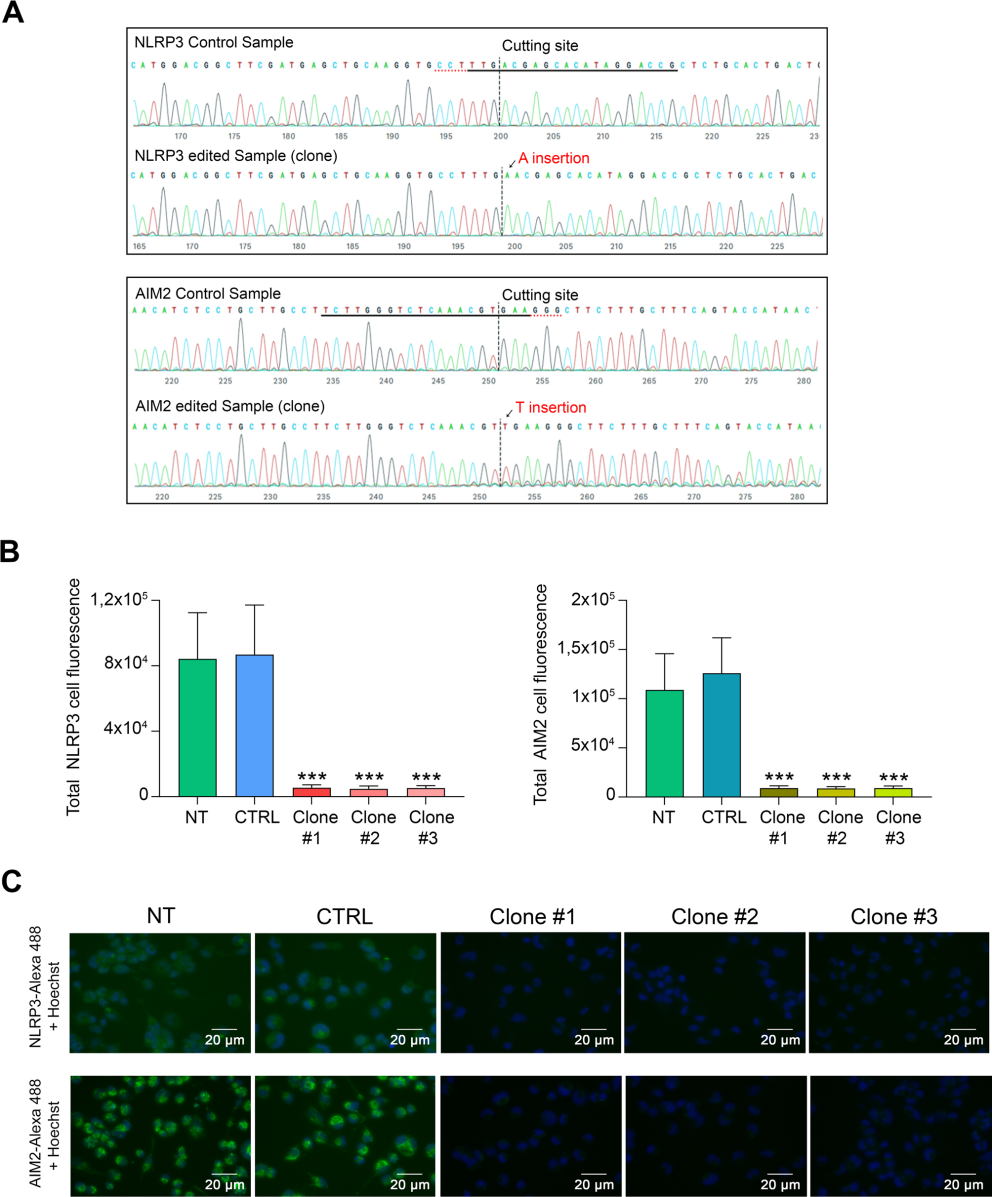
**A** Chromatogram illustrating the clonal and wild-type (control) sequences of the NLRP3 (top panel) and AIM2 (bottom panel) genes in the region around the Cas9 cutting site. The horizontal black underlined region represents the gRNA sequence, while the vertical black dotted line indicates the precise cutting site. Post-Cas9 cleavage DNA repair introduced an adenine insertion in the NLRP3 gene and a thymine insertion in the AIM2 gene immediately following the cleavage site followed by mixed sequencing bases. **B** Quantification of NLRP3 and AIM2 fluorescence intensity in non-transduced (NT), CTRL, and three different NLRP3 (left graph) or AIM2 (right graph) knockout clones of THP-1 cells. Fluorescence intensity was measured from at least 50 individual cells per condition in each experiment. **C** A representative image of each individual staining is shown, with Hoechst dye used to visualize the nuclei. Data represent the mean of three independent experiments. ***, *p* <.001 versus NT

To generate THP-1 cells lacking NLRP3 or AIM2 expression, the CRISPR/Cas9 system was employed. For this purpose, we utilized “all-in-one” lentiviral vectors, which encode both Cas9 and the gRNA within the same vector. The specific gRNA of NLRP3 gene targets an internal region of exon 3 (Fig. 1A, left panel) while the gRNA for the AIM2 gene targets an internal region of exon 4 (Fig. 1A, right panel). THP-1 cells were transduced with lentiviral particles containing the different vectors. Genomic DNA was then extracted from the transduced cells (bulk population) and sequenced to assess the knockout efficiency. Finally, to obtain a group of cells in which all were edited, i.e. 100% knockout, serial dilutions of the bulk population were performed and several clones, which by definition come from a single cell, were selected. The percentage of edited cells in these clones was analyzed and those in which 100% of the cells were edited were expanded (Fig. 1B). The editing efficiency for NLRP3 was approximately 60%, while AIM2 showed a higher editing efficiency, around 80% (Fig. 1C). The distribution of indels throughout the population of cells edited for NLRP3 showed some heterogeneity in DNA cleavage by Cas9 and subsequent repair by non-homologous end joining. Thus, predominantly single nucleotide insertions were generated in the majority of edited cells (approximately 70%) while deletions of 1, 5 or 11 nucleotides also were present, albeit in a much smaller proportion (Fig. 1D, left panel). In the case of AIM2, the distribution of indels was very similar to that obtained with NLRP3, showing predominance of single nucleotide insertion in 67% of the edited cells, and with deletions of 1, 6, 10 and 14 nucleotides in very low proportions (Fig. 1D, right panel). Subsequently, after analyzing the editing efficiency in different clones, three clonal populations were selected for each gene, all characterized by the insertion of a single nucleotide. Figure 2A shows the sequence of one of the selected clonal populations for NLRP3, where an adenine insertion occurred (Fig. 2A, top panel) and the sequence of a selected AIM2 clonal population with a thymine insertion (Fig. 2A, bottom panel). The insertion of a single nucleotide is expected to disrupt the open reading frame of the mRNA, resulting in the synthesis of a truncated (if the nucleotide insertion causes the appearance of a premature stop codon), elongated or non-functional protein. Using immunofluorescence, we were able to evidence a significant decrease in the expression levels of NLRP3 and AIM2 proteins in all selected clones (Fig. 2B and C).

### LPS and titanium ions modify the activation of NLRP3 and AIM2 inflammasomes

**Fig. 3:**
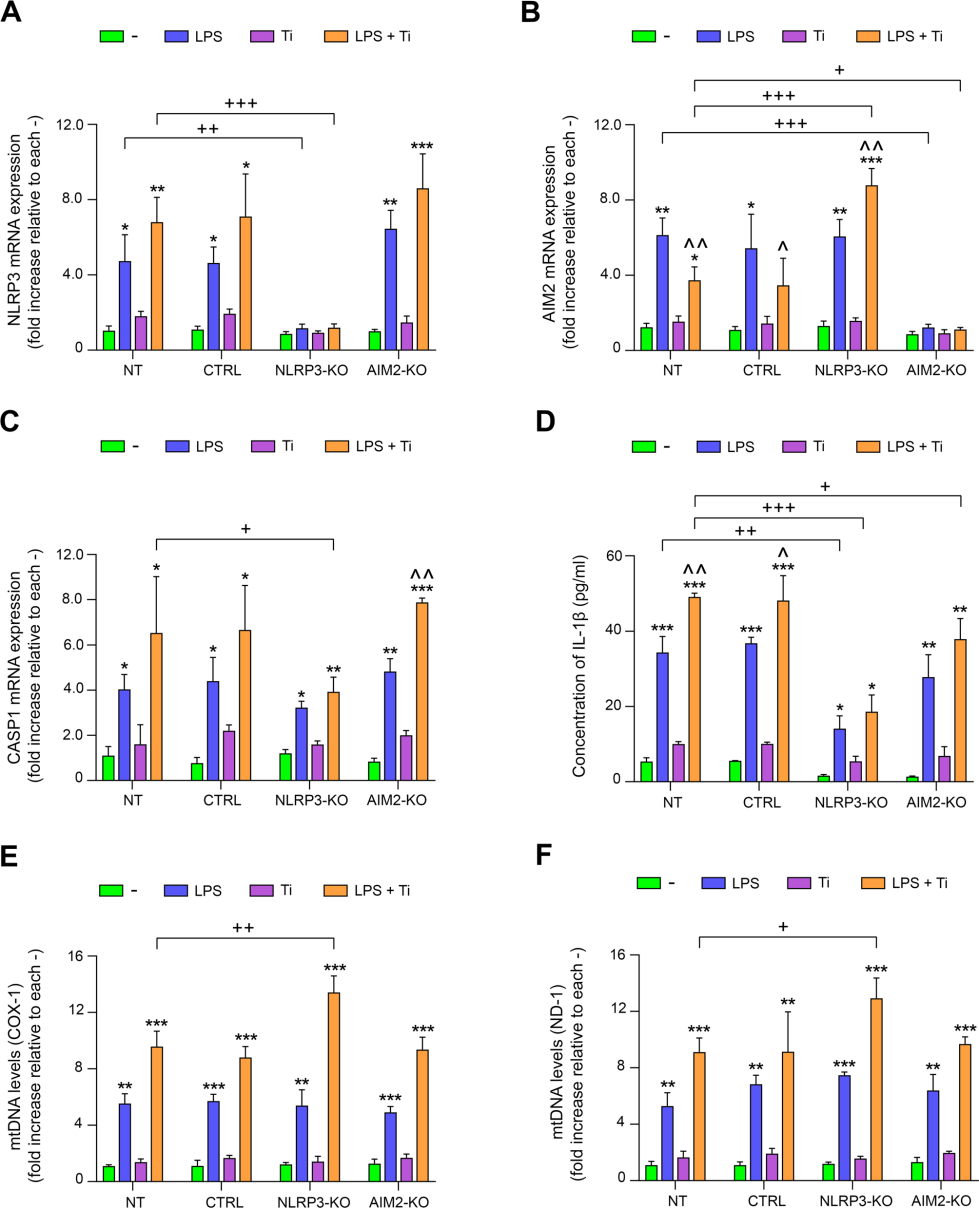
**A**, **B**, **C** NT, CTRL, NLRP3-KO and AIM2-KO THP-1 cells were treated with LPS, Ti or LPS + Ti and NLRP3 (**A**) AIM2 (**B**) and CASP1 (**C**) mRNA levels was analyzed by RT-qPCR. **D** Edited and non-edited THP-1 cells were treated with LPS, Ti or LPS + Ti and the levels of IL-1β were measured in supernatants by ELISA. **E**, **F** Mitochondrial DNA was extracted from the cytosolic fraction of NT, CTRL, NLRP3-KO, or AIM2-KO THP-1 cells, either untreated (-) or treated with LPS, Ti, or LPS + Ti. The levels of two mitochondrial genes (COX-1 and ND-2) were quantified using qPCR. Data are shown as mean (SD) of at least three independent experiments. **, *p* <.01; ***, *p* <.001 versus (-); ^, *p* <.05; ^^, *p* <.01 versus LPS; +, *p* <.05; ++, *p* <.01 NT versus KO among the treatments indicated by the square bracket. Although not shown in the graph to facilitate visualization, statistical analyses between CTRL and KO cells provided the same results as those between NT and KO cells

In order to evaluate the possible effects of bacterial components and metal ions on the activation of NLRP3 and AIM2 pathways in both edited and unedited THP-1 cells, both cell types were treated with PMA and then cultured with Ti in the presence or absence of LPS. First, NLRP3 expression levels were analyzed in edited and non-edited THP-1 cells by measuring messenger RNA (mRNA) levels. In non-edited cells, a significant increase in NLRP3 mRNA expression was observed following LPS treatment. This increase was even more pronounced when LPS was combined with Ti. Additionally, a slight upregulation of NLRP3 expression was detected in THP-1 cells treated with Ti alone. As expected, in NLRP3-KO cells, treatment with LPS and/or Ti had no effect. In contrast, AIM2-KO cells showed similar results to those observed in non-edited cells, with slightly higher NLRP3 mRNA levels (Fig. 3A). As previously explained in the introduction, so far it was only shown that AIM2 is activated only in the presence of dsDNA. Surprisingly, non-edited cells exhibited a strong increase in AIM2 mRNA levels when they were treated with LPS and simultaneously a significant decrease in mRNA levels was observed in cells treated with both LPS and Ti compared to those treated with LPS alone. Interestingly, this decrease in AIM2 levels observed in cells cultured with LPS and Ti was not seen in NLRP3-KO cells, where a significant increase in its expression was actually observed compared to the same cells treated with LPS alone. As expected, the presence of LPS and/or Ti had no effect on AIM2 mRNA levels in AIM2-KO cells (Fig. 3B).

Based on the observed differences in NLRP3 and AIM2 expression levels in response to bacterial components and/or titanium ions, we set out to test whether these variations were correlated with changes in the expression of the mediator Caspase 1 (CASP1). We found that indeed, CASP1 levels were significantly increased in unedited cells treated with LPS, and that increase was more pronounced in cells cultured with both LPS and Ti. In NLRP3-KO cells, the observed pattern was similar, but the increase in CASP1 mRNA levels was lower than in unedited cells for all treatments, with these differences being statistically significant in the case of LPS- and Ti-treated cells. In AIM2-KO cells, the results were similar to those observed in unedited cells and, as with NLRP3, CASP1 mRNA levels were slightly higher in these cells. It is further observed that CASP1 levels in AIM2-KO cells are higher than those in NLRP3-KO cells under LPS and LPS and Ti treatment conditions (Fig. 3C). Finally, we wanted to analyze the levels of IL-1β secreted by the different cell types under each treatment. Overall, both non-edited and edited cells showed a significant increase in IL-1β secretion when treated with LPS, with an even greater increase observed when Ti was also added. However, this increase was significantly lower in NLRP3-KO cells for both treatments compared to non-edited cells. Similarly, in AIM2-KO cells, IL-1β secretion was also significantly reduced when the cells were cultured with the combination of LPS and Ti compared with the same treatment in non-edited cells (Fig. 3D).

As previously mentioned, AIM2 can only be activated by cytosolic dsDNA (Fernandes-Alnemri et al. 2009). Consequently, any AIM2 activation in response to LPS should be considered an indirect effect. Our previous findings evidenced that LPS induces the release of mtDNA into the cytoplasm in mesenchymal stromal cells (MSCs) (Carrillo-Gálvez et al. 2024), so we analyzed mtDNA levels in THP-1 cells treated with LPS and/or Ti. As shown in Fig. 3E and F, LPS and LPS + Ti induced an increase in the amount of mtDNA released to the cytoplasm, estimated by qPCR in which mtDNA (but not mRNA) of COX-1 and ND-1 genes was used as a template in both edited and unedited cells. Interestingly, NLRP3-KO cells showed significantly higher mtDNA release with LPS + Ti than non-edited cells (Fig. 3E and F). This coincided with an increase in AIM2 expression in the same condition (Fig. 3B), suggesting a possible link between enhanced cytoplasmic mtDNA release and AIM2 upregulation in the absence of NLRP3.

### NLRP3 knockout reduces ROS production and pyroptosis induced by LPS and Ti, while AIM2 knockout only affects pyroptosis

**Fig. 4:**
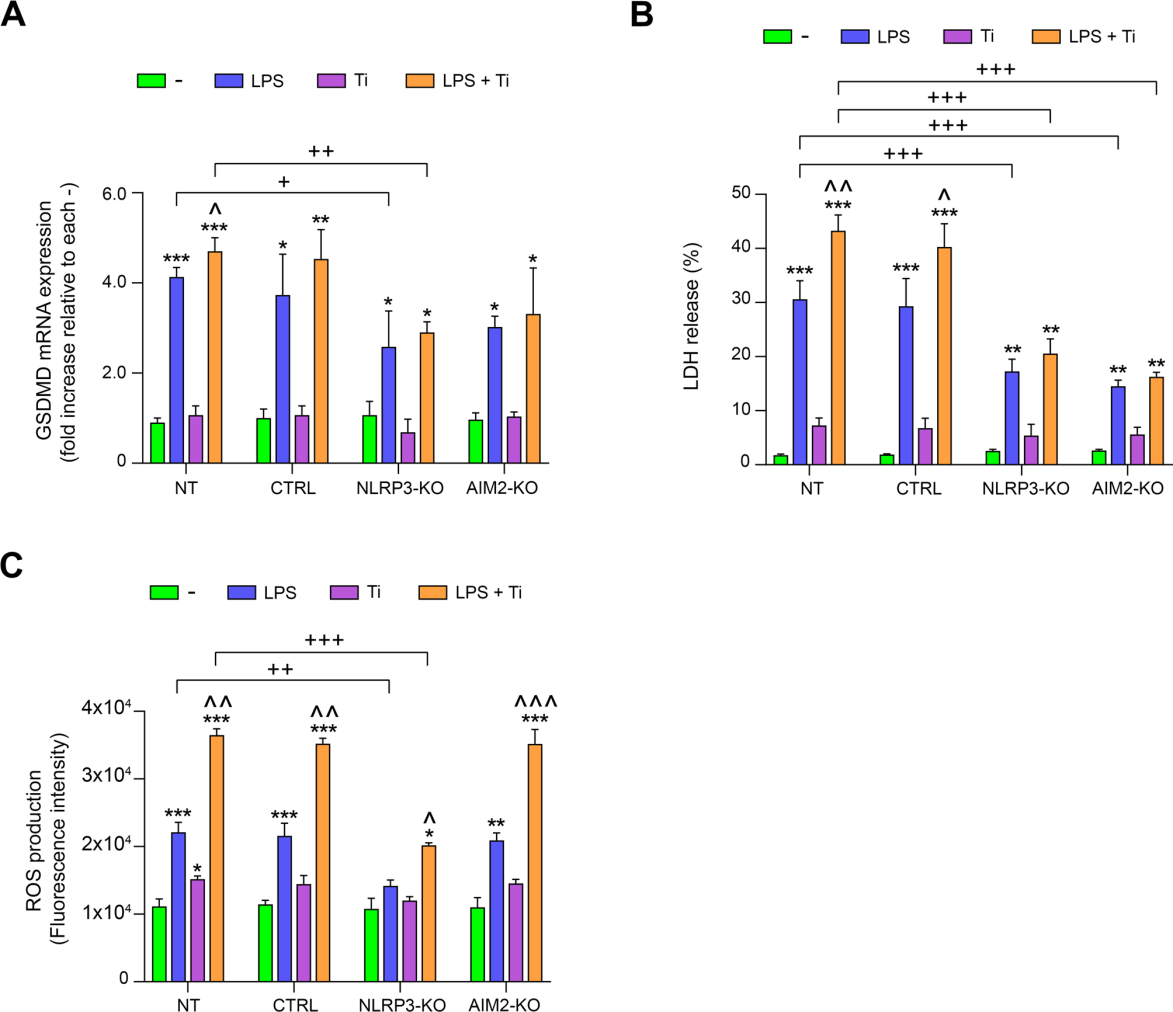
**A** Transduced THP-1 cells, including NT, CTRL, NLRP3-KO, and AIM2-KO, were treated with LPS, Ti, or LPS + Ti, and GSDMD mRNA levels was assessed via RT-qPCR. **B** Edited and non-edited THP-1 cells were treated with LPS, Ti, or LPS + Ti, and LDH secretion levels were quantified using a colorimetric assay. **C** Intracellular ROS levels were assessed in edited and non-edited THP-1 cells following treatment with LPS, Ti, or LPS + Ti using a fluorometric assay. Data are shown as mean (SD) of at least three independent experiments. *, *p* <.05; **, *p* <.01; ***, *p* <.001 versus (-); ^, *p* <.05; ^^, *p* <.01; ^^^, *p* <.001 versus LPS; +, *p* <.05; ++, *p* <.01; +++, *p* <.001 NT versus KO among the treatments indicated by the square bracket. Although not shown in the graph to facilitate visualization, statistical analyses between CTRL and KO cells provided the same results as those between NT and KO cells

We also aimed to investigate another process closely linked to inflammasome activation: pyroptosis. As mentioned in the introduction, pyroptosis is a type of cell death that occurs during the inflammatory response and is triggered by activation of the inflammasome (Marchesan et al. 2019). To this end, we analyzed the mRNA levels of GSDMD and measured the levels of LDH secreted by the cells. The amount of LDH released provides an indirect method to assess the process of pyroptosis (Rayamajhi et al. 2013). As observed, in non-edited cells, treatment with LPS significantly increased the expression levels of GSDMD as well as LDH release. This effect was further amplified when the cells were cultured with both LPS and Ti. In edited cells for both genes, the mRNA levels of GSDMD were lower compared to non-edited cells under any treatment condition. Importantly, this reduction was statistically significant when comparing LDH secretion levels between non-edited and edited cells (Fig. 4A and B). ROS are well known to induce both NLRP3 activation (Liu et al. 2022; Dominic et al. 2022) and the pyroptotic process (Wang et al. 2024). Therefore, we analyzed ROS production in THP-1 cells to evaluate their potential contribution to the observed inflammasome activation. A significant increase in ROS production was observed in both unedited THP-1 and AIM2-KO cells cultured in the presence of LPS. Again, this production was even higher when the cells were treated with both LPS and Ti. However, in NLRP3-KO cells, no significant differences in ROS production were observed between untreated and LPS-treated cells, although these differences became statistically significant when cells were cultured with both LPS and Ti.

### ROS could be responsible for the enhanced AIM2 activation in NLRP3-KO cells treated with LPS and Ti

**Fig. 5:**
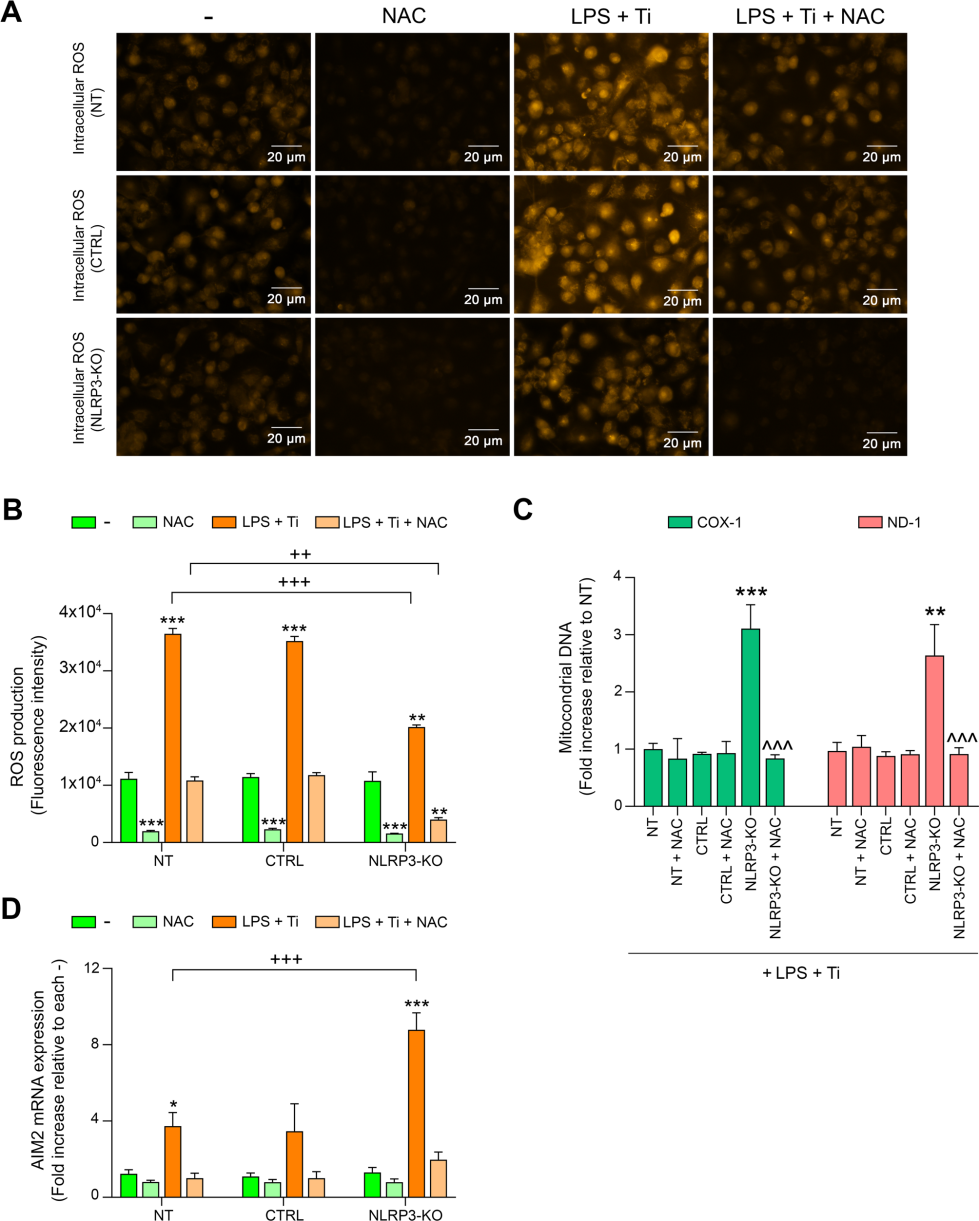
**A** Representative images showing fluorescence intensity of intracellular ROS in NT, CTRL, and NLRP3-KO cells, either untreated (-) or treated with NAC, LPS + Ti, or LPS + Ti + NAC. **B** Intracellular ROS levels were evaluated in NT, CTRL, and NLRP3-KO THP-1 cells, either untreated (-) or treated with NAC, LPS + Ti, or LPS + Ti + NAC, using a fluorometric assay. **C** Mitochondrial DNA was isolated from the cytosolic fraction of NT, CTRL, and NLRP3-KO cells, either untreated (-) or treated with NAC, LPS + Ti, or LPS + Ti + NAC. The levels of two mitochondrial genes (COX-1 and ND-2) were quantified by qPCR. **D** AIM2 mRNA levels was analyzed by RT-qPCR in NT, CTRL, and NLRP3-KO THP-1 cells untreated (-) or treated with NAC, LPS + Ti, or LPS + Ti + NAC. Data are shown as mean (SD) of at least three independent experiments. *, *p* <.05; **, *p* <.01; ***, *p* <.001 versus (-); ^^^, *p* <.001 versus NLRP3-KO; ++, *p* <.01; +++, *p* <.001 NT versus KO among the treatments indicated by the square bracket. Although not shown in the graph to facilitate visualization, statistical analyses between CTRL and KO cells provided the same results as those between NT and KO cells

Based on the unexpected findings regarding the variation in AIM2 mRNA levels and the differences in mtDNA release into the cytoplasm between unedited and NLRP3-KO THP-1 cells treated with LPS and Ti, we aimed to investigate the potential causes of these changes in more detail. It is well documented that ROS are important inducers of mitochondrial damage (Kowaltowski et al. 1999; Song et al. 2024), which can lead to mitochondrial membrane destabilization and subsequent release of the internal contents of mitochondria, including DNA, into the cytoplasm. As shown in Fig. 5A and B, ROS levels were significantly reduced in NLRP3-KO macrophages compared to WT cells upon LPS and Ti stimulation (Fig. 5A and B). Interestingly, despite this reduction, a marked increase in AIM2 expression was observed in this KO cells (Fig. 5D). This apparent discrepancy led us to hypothesize that even the residual ROS levels present in the absence of NLRP3 might still be sufficient to induce mitochondrial stress and trigger the release of mtDNA into the cytosol. Indeed, we detected increased levels of cytosolic mtDNA in NLRP3-KO cells compared to WT cells under the same conditions (Fig. 5C). To further investigate the role of ROS in this process, we treated NLRP3-KO cells with the antioxidant NAC. This treatment resulted in a notable reduction in both cytosolic mtDNA (Fig. 5C) and AIM2 expression (Fig. 5D), supporting the idea that mitochondrial ROS, even at submaximal levels, may be sufficient to promote mtDNA release and AIM2 activation. These findings suggest that the threshold of ROS required to initiate AIM2 activation through mtDNA release may be relatively low, and that this pathway may become more prominent in the absence of NLRP3.

### The absence of NLRP3 and AIM2 modifies M1/M2 polarization in macrophages exposed to LPS and Ti

**Fig. 6:**
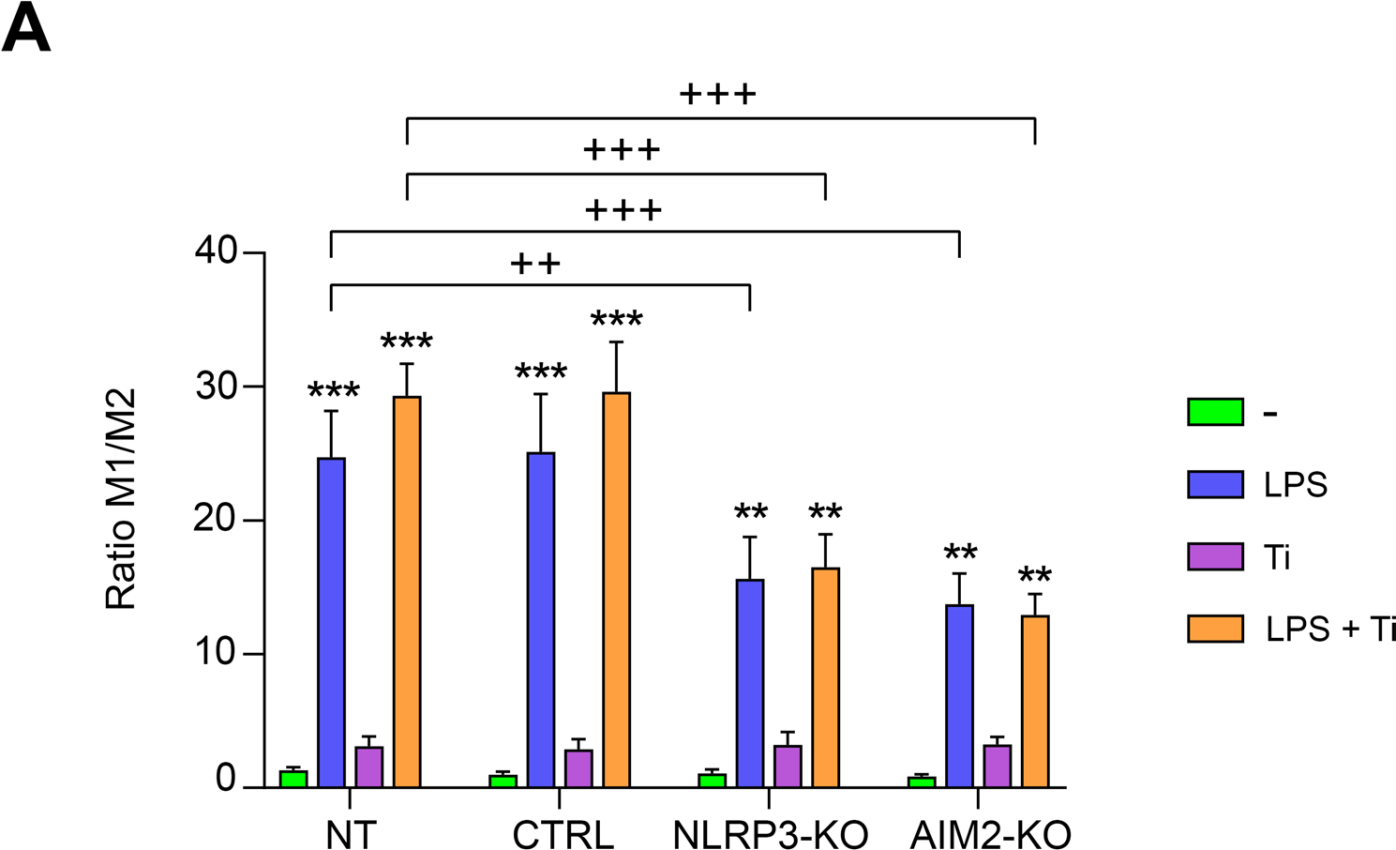
**A** Transduced THP-1 cells, including NT, CTRL, NLRP3-KO, and AIM2-KO, were treated with LPS, Ti, or LPS + Ti. The mRNA levels of three M1 macrophage-specific markers (CXCL9, CXCL10, and IRF-1) and three M2 macrophage-specific markers (CCL17, ALOX15, and MRC1) were analyzed by RT-qPCR. The M1/M2 ratio was calculated as the average expression of M1 genes relative to the average expression of M2 genes. Data are shown as mean (SD) of at least three independent experiments. **, *p* <.01; ***, *p* <.001 versus (-) ++, *p* <.01; +++, *p* <.001 NT versus KO among the treatments indicated by the square bracket. Although not shown in the graph to facilitate visualization, statistical analyses between CTRL and KO cells provided the same results as those between NT and KO cells

Finally, we wanted to investigate if the absence of NLRP3 or AIM2 inflammasomes affects macrophage polarization towards pro-inflammatory or anti-inflammatory state in an inflammatory environment. For this purpose, we analyzed the M1/M2 ratio in edited and unedited cells subjected to all treatments. We observed that both LPS and LPS + Ti induced a pro-inflammatory (M1) phenotype in all cell types, as the ratio was significantly higher than 1 in all cases. However, it is important to note that this ratio was significantly lower in edited cells compared to unedited cells (Fig. 6A).

## Discussion

Current treatments for both periodontitis and peri-implantitis are almost exclusively limited to antibiotics. However, bacterial elimination has proven insufficient for long-term treatment, largely due to the growing antibiotic resistance of periodontal pathogens (Haque et al. 2022). Furthermore, in peri-implantitis, it is crucial to consider not only the bacterial component but also the titanium particles and ions released from the implant surface, which can have pro-inflammatory effects. While often considered similar, clear evidence shows that peri-implantitis and periodontitis differ pathophysiologically. For instance, peri-implantitis biopsies show a higher immune cell infiltration (Carcuac and Berglundh 2014), and bone resorption is more pronounced compared to periodontitis (Derks et al. 2016). New therapies targeting the immune response are crucial, given its key role in these chronic inflammatory disorders. In that sense, the objective of this study was to examine the inflammatory process in THP-1 cell derived macrophages, specifically targeting NLRP3 and AIM2 inflammasome pathways, under exposure to bacterial components (LPS), titanium ions, or their combination.

LPS is known to increase mRNA and protein levels of NLRP3, CASP1, and IL-1β in innate immune cells *in vitro* (Mezzasoma et al. 2023; Seo et al. 2023; He et al. 2012). Although studies using titanium ions are limited, they have shown NLRP3 activation in macrophages (Pettersson et al. 2022) and T cells (Li et al. 2020), with a stronger effect when combined with bacterial components (Pettersson et al. 2017). Our findings are consistent with these studies, strongly suggesting a synergistic interaction between metal ions and bacterial components in the activation of the inflammatory process in macrophages. In addition, in this report we show for the first time an indirect induction of AIM2 in the presence of LPS and how titanium can modulate the activation of this signaling pathway in macrophages. Our findings suggest that AIM2 induction in response to LPS is likely driven by the presence of cytoplasmic mtDNA. Notably, consistent with our previous research in MSCs (Carrillo-Gálvez et al. 2024), we have observed a reduction in AIM2 expression in cells treated with LPS + Ti compared to LPS alone. This supports the idea that when metallic component is present, NLRP3 pathway predominates over AIM2 pathway.

As we have observed in THP-1 derived macrophages, it is well established that NLRP3 and AIM2 are upregulated in saliva (Arunachalam et al. 2024), periapical lesions (Ran et al. 2017; Guan et al. 2020) and gingival tissues (Xue et al. 2015) of periodontitis patients, driving an increase in IL-1β secretion. Although the impact of titanium on peri-implantitis progression has been widely studied (Asa’ad et al. 2022; Chen et al. 2023), there is limited research on the molecular mechanisms underlying this effect. Specifically, there are very few studies that analyze inflammasomes in peri-implantitis patient samples. Ganesan et al. investigated the expression of other inflammasomes, such as NLRP2, NLRP8, and NLRP12 in periodontitis samples (Ganesan et al. 2022) and, recently, our research group demonstrated that the chronic inflammation observed in peri-implantitis patients could partly be attributed to the activation of the NLRP3 and AIM2 signaling pathways (Galindo-Moreno et al. 2023). This activation can be, in fact, correlated with the presence of specific bacteria in the environment (Padial-Molina et al. 2024).

In vitro, numerous studies are investigating the impact of NLRP3 expression suppression in various cell lines. To this end, different inhibitors such as dopamine, CY-9, or anthracycline have been used. These studies have shown that inhibiting NLRP3 leads to a reduction in IL-1β secretion levels in macrophages (Jiang et al. 2017; Yan et al. 2015; Köse-Vogel et al. 2019), however, the pyroptosis process remained unaffected (Köse-Vogel et al. 2019). Regarding studies in which the expression of NLRP3 gene has been abolished, there are few in the literature. Busch et al. analyzed THP-1 NLRP3-KO cells treated with LPS, observing reduced inflammatory responses (Busch et al. 2022a). They also noted a similar decrease when these cells were exposed to micro- and nanoplastics (Busch et al. 2022b). In our previous publication, we demonstrated that knocking out NLRP3 and AIM2 expression reduces IL-1β production and pyroptosis in alveolar bone-derived MSCs (Carrillo-Gálvez et al. 2024). To our knowledge, there are no further studies in which AIM2 expression has been suppressed to see its effect on the inflammatory process.

In this report, we have generated loss-of-function mutants for both genes in THP-1 cells. We consider it important to perform knockout rather than using inhibitors, as inhibitors block already expressed proteins, avoiding the analysis of potential compensatory mechanisms in response to gene deletion or mutual regulation between different signaling pathways. According with findings from the previously cited studies, NLRP3 and AIM2 KO THP-1 derived macrophages, significantly reduces the secretion of active IL-1β compared to NT and CTRL cells in the presence of LPS alone or in combination with Ti. This effect is more pronounced in NLRP3-KO cells.

In this study, we also analyzed ROS production by these cells, given their well-established relevance in these signaling pathways. It is known that NLRP3 activation enhances ROS production, which in turn triggers NLRP3 activation, creating a positive feedback loop that amplifies the inflammatory response (Dominic et al. 2022; Abais et al. 2015). Consistently, we observed a significant reduction in ROS production in NLRP3-KO cells, whereas ROS levels in AIM2-KO cells were similar to those observed in unedited cells.

Finally, we also analyzed the process of pyroptosis. As previously mentioned, a study in macrophages, in which NLRP3 expression was inhibited, reported no alteration in the pyroptosis process (Köse-Vogel et al. 2019). However, our research shows opposite results, as we observed a decrease in GSDMD mRNA levels in NLRP3- and AIM2-KO cells treated with LPS or LPS + Ti. Furthermore, LDH secretion levels were significantly reduced in both KO cell lines under these treatments compared to unedited cells.

Inflammation is a highly regulated process and it is important to take into account the possibility that there is cross regulation between different signaling pathways. Specifically, while AIM2 expression decreases in NT cells treated with LPS + Ti compared to LPS alone, which could indicate a suppressive effect of Ti on AIM2 expression, this effect is reversed in NLRP3-KO cells, where AIM2 expression increases under LPS + Ti conditions. These findings suggest that the presence of titanium favors NLRP3 pathway activation, which may functionally outcompete AIM2 when both are present. The upregulation of AIM2 in the absence of NLRP3 under LPS + Ti further supports this hypothesis, highlighting the importance of titanium as a modulator of inflammasome pathway dynamics. To further explore the underlying mechanism driving the increased AIM2 expression in NLRP3-KO cells treated with LPS and Ti, we focused on the potential role of ROS as a previous study had shown that mitochondrial ROS can indirectly activate AIM2 (Crane et al. 2014). Based on these findings, we hypothesized that in absence of NLRP3, ROS generated by LPS and Ti, unable to interact with NLRP3, might promote AIM2 activation through increased release of mtDNA into the cytoplasm. Supporting this hypothesis, we observed elevated cytosolic mtDNA and AIM2 expression in NLRP3-KO cells, both of which were reduced upon ROS inhibition. To the best of our knowledge, this is the first time that a potential reciprocal regulation of the activation of two inflammasomes has been shown as a compensatory mechanism to maintain a pronounced inflammatory response under conditions of inflammation induced by bacterial and/or metallic components.

Finally, we also examined the polarization of THP-1-derived macrophages toward a pro-inflammatory (M1) or anti-inflammatory (M2) phenotype by analyzing the M1/M2 ratio in both edited and unedited macrophages. This analysis is crucial given the importance of macrophage polarization in either amplifying or resolving the inflammatory response in periodontitis and peri-implantitis (Mo et al. 2024; Li et al. 2024). Our results show significant polarization toward pro-inflammatory macrophages (M1) with LPS or LPS + Ti. These findings are in agreement with previous studies showing that activation of the NLRP3 inflammasome promotes M1 macrophage polarization in inflammatory environments (Zhang et al. 2020; Wisitpongpun et al. 2022). In NLRP3-KO cells, although polarization toward M1 remains evident, the ratio is significantly lower compared to unedited cells. This contrasts with a previous study in which the authors showed that inflammasome inhibition increased the M1/M2 ratio by reducing IL-4 secretion (Strizova et al. 2023). Similarly, AIM2 has also been reported to favor M1 polarization while suppressing M2 markers in renal carcinoma (Chai et al. 2021). Interestingly, while the reduction in IL-1β was more pronounced in NLRP3-KO cells, the M1/M2 ratio was slightly lower in AIM2-KO cells. This observation suggests that AIM2 may contribute to the regulation of the inflammatory response, potentially through non-canonical pathways. However, we acknowledge that other factors, such as differences in the timing or kinetics of marker expression, or indirect effects mediated by cytokines like IL-1β, could also influence these results. Therefore, a more detailed characterization would be necessary to fully understand the mechanisms involved.

**Fig. 7:**
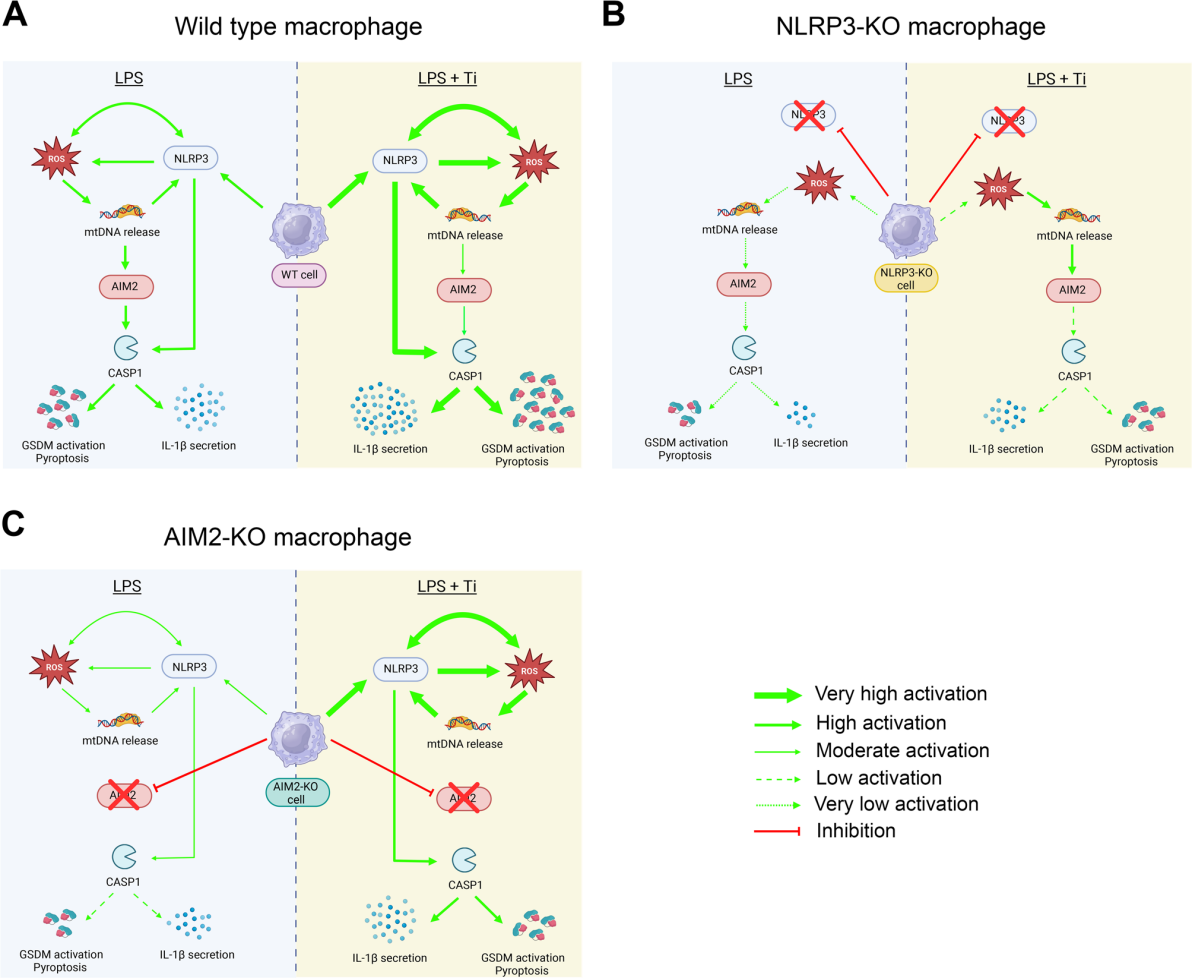
Proposed model summarizing the role of NLRP3 and AIM2 inflammasomes in macrophages under periodontitis (LPS) and peri-implantitis (LPS + Ti) conditions. **A** In WT macrophages, LPS induces NLRP3 activation, triggering ROS production, mtDNA release, AIM2 activation, IL-1β secretion, and pyroptosis. LPS + Ti amplifies NLRP3 activation, reduces AIM2 activation, but further increases IL-1β secretion and pyroptosis. **B** In NLRP3-KO macrophages, the absence of the feedback loop reduces ROS and IL-1β levels, although LPS + Ti partially restores ROS and AIM2 activation, slightly increasing IL-1β secretion and pyroptosis compared to LPS alone. **C** In AIM2-KO macrophages, NLRP3 activation by LPS increases IL-1β secretion and pyroptosis, but both remain lower than in WT cells. LPS + Ti strongly activates NLRP3, but without AIM2, IL-1β and pyroptosis remain limited. Arrow thickness and intensity reflect the relative degree of inflammasome activation observed under each condition. Figure created with BioRender.com

Based on our results, we propose a model where, in wild-type (WT) macrophages, LPS (periodontitis scenario) increases NLRP3 activation, driving ROS production. These ROS enhance NLRP3 expression through a positive feedback loop and induce mtDNA release, activating AIM2. This culminates in elevated IL-1β secretion and pyroptosis. The presence of LPS + Ti (peri-implantitis scenario) amplifies the effect induced by NLRP3 and, although it reduces AIM2 activation, the final outcome is a further increase in IL-1β levels and pyroptosis (Fig. 7A). On the other hand, in NLRP3-KO macrophages, the absence of the feedback loop reduces ROS levels and, although they maintain the ability to induce mtDNA release and activate AIM2, IL-1β secretion and pyroptosis are significantly decreased in both periodontitis and peri-implantitis scenarios. However, in this last scenario (LPS + Ti), titanium enhances ROS production, inducing a greater AIM2 activation. This results in a slight increase in IL-1β activation and pyroptosis, although at a lower level than in WT cells (Fig. 7B). Finally, in AIM2-KO macrophages, LPS activates only NLRP3, leading to increased IL-1β secretion and pyroptosis, but at lower levels than in WT cells. In peri-implantitis, LPS + Ti strongly activates NLRP3, but without AIM2 activation, IL-1β and pyroptosis remain lower than in WT cells (Fig. 7C).

These findings could be highly significant, not only for gaining a deeper understanding of the regulation of the inflammatory process but also because these results could help to identify new therapeutic targets aimed at modulating the immune response in patients with periodontitis, peri-implantitis and other inflammatory/autoimmune diseases.

## Conclusions

Our findings highlight the critical role of titanium in exacerbating inflammation in environments where metal interaction may occur, such as around dental implants or functional prostheses, as the combination of bacterial and metallic components amplifies IL-1β secretion and pyroptosis. For the first time, we show that NLRP3 and AIM2 inflammasomes are mutually regulated, i.e. the absence of one modulates the activation of the other. We also reveal that ROS play a key role in the indirect activation of AIM2 in response to LPS or LPS + Ti. Lastly, we show that inflammasomes significantly influence macrophage polarization, a crucial factor in resolving inflammation. These results provide valuable insights for developing novel therapeutic strategies for these or others inflammatory diseases.

## Data Availability

No datasets were generated or analysed during the current study.

## Abbreviations

AIM2: Absent in melanoma 2
Cas9: Crispr associated protein 9
CASP1: Caspase-1
CRISPR: Clustered Regularly Interspaced Short Palindromic Repeats
DAMPs: Danger associated molecular pattern
dsDNA: Double-stranded DNA
gRNA: Guide-RNA
GSDMD: Gasdermin-D
IL-1β: Interleukin-1β
IL-18: Interleukin-18
KO: Knockout
LDH: Lactate dehydrogenase
LPS: Lipopolysaccharide
MSCs: Mesenchymal stromal cells
mtDNA: Mitochondrial DNA
mRNA: Messenger RNA
NAC: N-Acetyl-L-cysteine
NLRP3: NLR family pyrin domain containing 3
PAMPs: Pathogen associated molecular pattern
PMA: Phorbol 12-myristate-13-acetate
ROS: Reactive oxygen species
RT-qPCR: Reverse transcription polymerase chain reaction
Ti: Titanium ions
